# Building a machine learning-assisted echocardiography prediction tool for children at risk for cancer therapy-related cardiomyopathy

**DOI:** 10.1186/s40959-024-00268-4

**Published:** 2024-10-09

**Authors:** Lindsay A. Edwards, Christina Yang, Surbhi Sharma, Zih-Hua Chen, Lahari Gorantla, Sanika A. Joshi, Nicolas J. Longhi, Nahom Worku, Jamie S. Yang, Brandy Martinez Di Pietro, Saro Armenian, Aarti Bhat, William Border, Sujatha Buddhe, Nancy Blythe, Kayla Stratton, Kasey J. Leger, Wendy M. Leisenring, Lillian R. Meacham, Paul C. Nathan, Shanti Narasimhan, Ritu Sachdeva, Karim Sadak, Eric J. Chow, Patrick M. Boyle

**Affiliations:** 1 Department of Pediatrics, Division of Cardiology, Duke University Medical Center, DUMC Box 3090, Durham, NC 27710 USA; 2 Department of Pediatrics, University of Washington, Seattle, WA USA; 3 Children’s National Medical Center, Washington, DC USA; 4 Department of Bioengineering, University of Washington, 3720 15th Ave NE N361, UW Mailbox 355061, Seattle, WA 98195 USA; 5 Seattle Children’s Hospital, Seattle, WA USA; 6 Departments of Pediatrics and Population Sciences, City of Hope, Duarte, CA USA; 7 Department of Pediatrics, Emory University School of Medicine, Atlanta, GA USA; 8 Division of Pediatric Cardiology, Stanford University School of Medicine, Palo Alto, CA USA; 9 Clinical Research and Public Health Sciences Divisions, Fred Hutchinson Cancer Center, Seattle, WA USA; 10 Department of Pediatrics, University of Toronto, The Hospital for Sick Children, Toronto, ON Canada; 11 Department of Pediatrics, University of Minnesota, Masonic Children’s Hospital, Minneapolis, MN USA; 12 Institute for Stem Cell and Regenerative Medicine, University of Washington, Seattle, WA USA; 13 Center for Cardiovascular Biology, University of Washington, Seattle, WA USA

**Keywords:** Machine learning, Cardiomyopathy, Cancer survivorship, Echocardiography

## Abstract

**Background:**

Despite routine echocardiographic surveillance for childhood cancer survivors, the ability to predict cardiomyopathy risk in individual patients is limited. We explored the feasibility and optimal processes for machine learning-enhanced cardiomyopathy prediction in survivors using serial echocardiograms from five centers.

**Methods:**

We designed a series of deep convolutional neural networks (DCNNs) for prediction of cardiomyopathy (shortening fraction ≤ 28% or ejection fraction ≤ 50% on two occasions) for at-risk survivors ≥ 1-year post initial cancer therapy. We built DCNNs with four subsets of echocardiographic data differing in timing relative to case (survivor who developed cardiomyopathy) index diagnosis and two input formats (montages) with differing image selections. We used holdout subsets in a 10-fold cross-validation framework and standard metrics to assess model performance (e.g., F1-score, area under the precision-recall curve [AUPRC]). Performance of the input formats was compared using a combined 5 × 2 cross-validation F-test.

**Results:**

The dataset included 542 pairs of montages: 171 montage pairs from 45 cases at time of cardiomyopathy diagnosis or pre-diagnosis and 371 pairs from 70 at-risk survivors who didn’t develop cardiomyopathy during follow-up (non-case). The DCNN trained to distinguish between non-case and time of cardiomyopathy diagnosis or pre-diagnosis case montages achieved an AUROC of 0.89 ± 0.02, AUPRC 0.83 ± 0.03, and F1-score: 0.76 ± 0.04. When limited to smaller subsets of case data (e.g., ≥ 1 or 2 years pre-diagnosis), performance worsened. Model input format did not impact performance accuracy across models.

**Conclusions:**

This methodology is a promising first step toward development of a DCNN capable of accurately differentiating pre-diagnosis versus non-case echocardiograms to predict survivors more likely to develop cardiomyopathy.

**Graphical Abstract:**

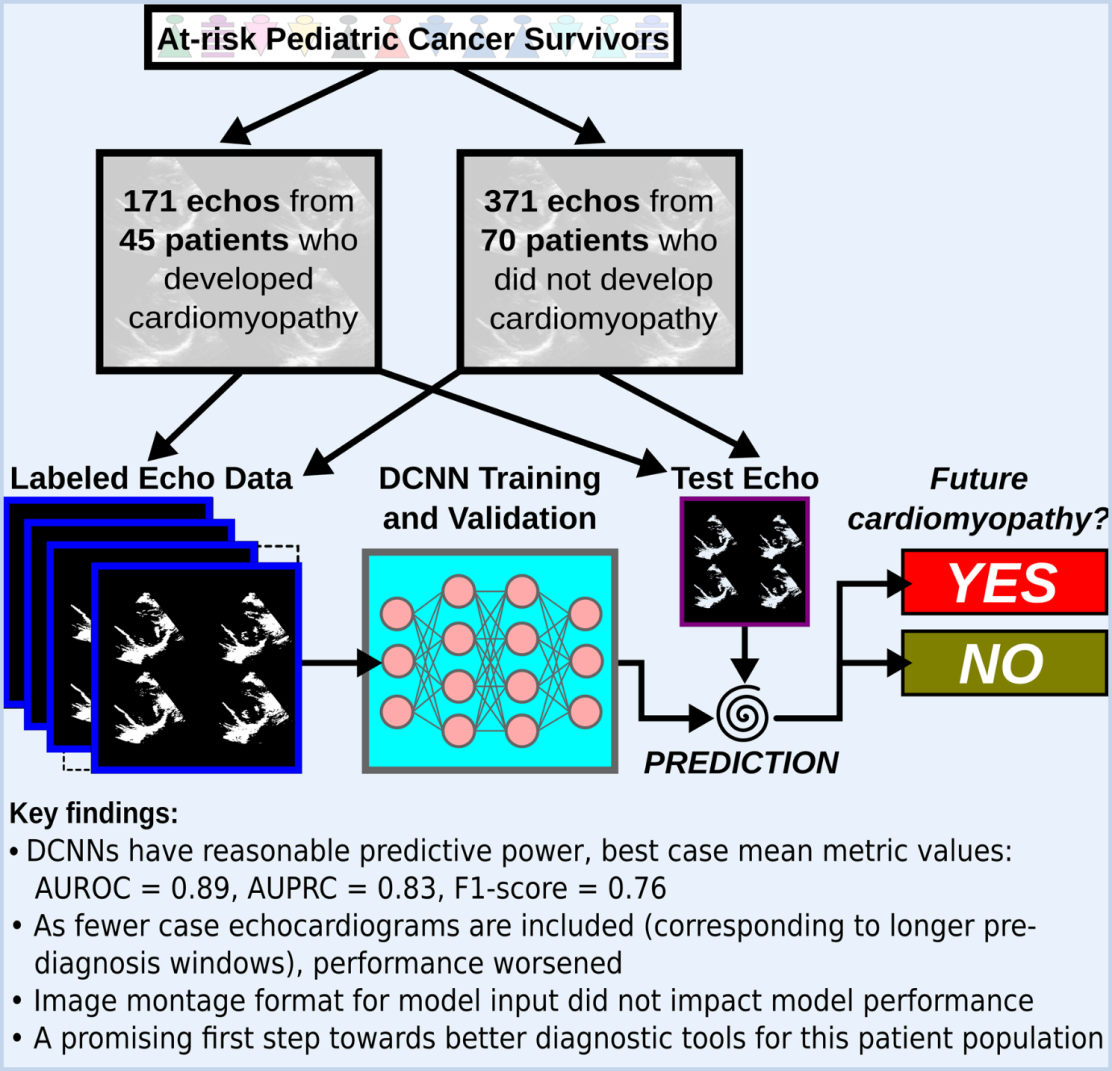

**Supplementary Information:**

The online version contains supplementary material available at 10.1186/s40959-024-00268-4.

## Background

Treatment-related cardiomyopathy is a leading cause of premature morbidity in childhood cancer survivors [1, 2]. Although there is insufficient evidence to guide cardiomyopathy management in pediatric cancer survivors, the pediatric and adult-based evidence for non-cancer-related cardiomyopathy suggests that earlier intervention can mitigate or delay cardiomyopathy progression [3–5]. Currently, national and international consensus guidelines recommend the routine use of 2-dimensional (2D) echocardiography to screen childhood cancer survivors at elevated risk for cardiomyopathy (due to anthracycline chemotherapy or chest radiation exposure) for signs of cardiomyopathy including left ventricular (LV) systolic dysfunction and changes in LV geometry [6, 7]. LV shortening fraction (SF) and ejection fraction (EF), the most commonly used criteria for defining therapy-related cardiomyopathy in children, have limited sensitivity, largely due to inter- and intra-observer variability and load dependence [1, 8]. Additionally, the screening utility of LV SF and EF is limited; by the time significant changes in these systolic functional parameters are noted, irreversible cardiac remodeling has likely occurred, and it may be too late to alter long-term cardiac outcome [9]. Therefore, methods that improve the detection of cardiac remodeling and predict progression to cardiomyopathy in childhood cancer survivors may have important clinical implications.

Deep learning, a subfield of machine learning, involves the use of neural networks (computer algorithms designed to mimic processing by the human brain) to automatically extract patterns from large unstructured datasets, such as medical images, and is increasingly utilized in medicine for disease diagnosis, onset, and outcome prediction. A deep convolutional neural network (DCNN) is an elaborate form of neural network that provides superior performance for complex tasks such as image processing but requires more extensive training and computational power. In this pilot study, we used retrospective data collected at five North American sites to evaluate the feasibility of and optimal processes for DCNN prediction of cardiomyopathy in childhood cancer survivors. The goals of this study were to (1) identify the optimal imaging data input format for the DCNN and (2) to evaluate feasibility of a DCNN tasked with prediction of cardiomyopathy several years before diagnosis by observing model performance as dataset size decreased. This work will guide future efforts that leverage echocardiographic data from a much larger research consortium, the Children’s Oncology Group (COG), with > 200 institutions, primarily in the United States and Canada.

## Methods

### Study population

An extant, retrospective dataset including individuals diagnosed with cancer at age < 21 years and cared for at one of five centers (Children’s Healthcare of Atlanta, City of Hope Medical Center, The Hospital for Sick Children, Seattle Children’s Hospital, and University of Minnesota Masonic Children’s Hospital) was used for model development and testing [10]. All participants were childhood cancer survivors at-risk for cardiomyopathy due to cardiotoxic cancer therapy exposure who survived at least one year past completion of initial cancer treatment. Cardiomyopathy cases were defined as at-risk survivors with quantitatively decreased systolic function with SF ≤ 28% or EF ≤ 50% on at least two clinical 2D echocardiograms obtained as part of routine surveillance, with at least one study performed following cancer therapy completion, or if these quantitative echocardiographic criteria were met on only one occasion but chronic heart failure therapy was subsequently initiated. Non-cases from all five sites were defined as at-risk (i.e., anthracycline and/or chest radiotherapy exposed) survivors who underwent routine echocardiographic surveillance and did not develop cardiomyopathy during the available follow-up period. Specifically, non-cases had to have SF ≥ 30% and EF ≥ 55% and could not have qualitative changes concerning for cardiomyopathy reported on any of their institutional echocardiographic reports. Exclusion criteria that applied to both cardiomyopathy cases and non-cases included any individual with congenital heart disease and or a genetic syndrome associated with cardiac disease (e.g., Trisomy 21). For all cases and non-cases, research staff at each institution abstracted relevant demographic and clinical data into a shared central Research Electronic Data Capture (REDCap) database [11, 12] and uploaded deidentified (except for a study-specific ID) echocardiograms in DICOM format to PICOM365, a secure cloud-based server (SciImage, Los Altos, CA). This study was approved by the IRBs of all five institutions with waiver of consent as all data were ascertained from the existing medical record.

### Data selection and processing

Echocardiogram video clips in the parasternal short axis (PSAX) view at the level of the papillary muscle in grayscale or other color ranges (e.g., sepia) were manually acquired by a study physician or sonographer. PSAX was selected as it is the recommended view for assessing LV size and wall thickness in children [13]. Images obtained in other echocardiographic views, with operator sweeping/fanning away from the papillary muscles, without electrocardiogram (ECG) tracing or with uninterpretable ECG tracing, and with objects such as cursors or prominent ECG tracings obscuring the cardiac structures were excluded. Of note, poor quality images due to technical limitations such as poor acoustic windows or lung artifact were not excluded to ensure the dataset reflected real-world data.

**Fig. 1:**
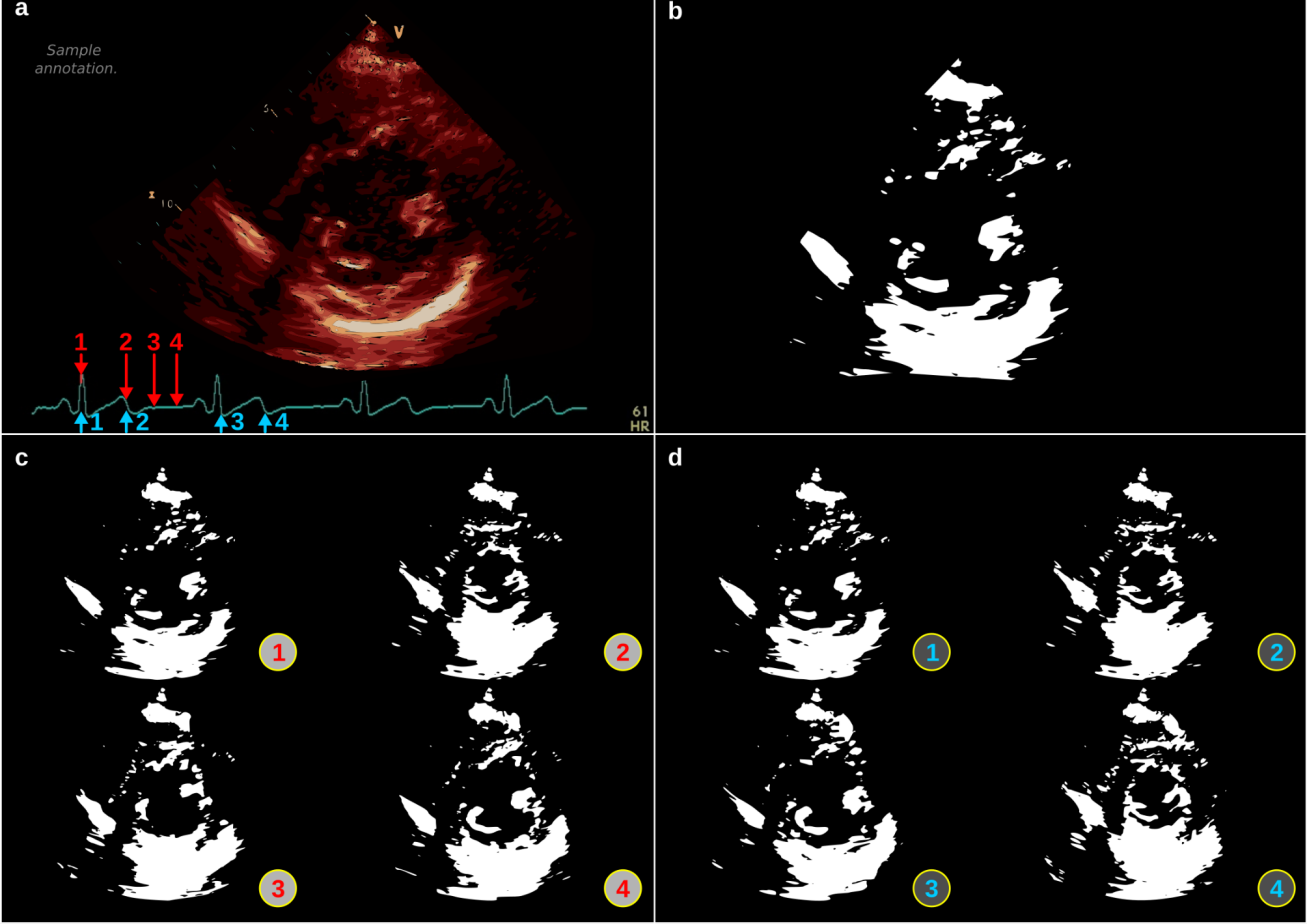
Image preprocessing and montages. **a** Illustration of an unprocessed image with sepia colorization and annotations. Superimposed arrows on ECG indicate where frames were extracted for montage Type 1 (red) and 2 (blue). **b** Processed image. **c** Montage Type I with frames corresponding to four points in a single cardiac cycle: **1** the R wave peak; **2** one-third of the R-R interval, approximating the end of the T wave; **3** one-half the R-R, approximating the middle of the TP segment, and **4** two-thirds the R-R interval, approximating the onset of the next P wave. **d** Montage Type 2 with numbered frames corresponding to four points in two consecutive cardiac cycles: **1** first R wave peak; **2** one-third of the first R-R interval, approximating the end of the first T wave; **3** second R wave peak; **4** one-third of the second R-R interval, approximating the end of the second T wave

One PSAX video clip per study was randomly selected, and the DICOM data was downloaded from the cloud-based server. Four individual still frames from the video clip were identified based on timing with respect to the ECG tracing and extracted (Fig. 1). We opted to use frame subsets rather than full video clips for two reasons: (1) using fewer images dramatically reduced the computational burden of DCNN training and validation; and, (2) selecting frames according to ECG landmarks helped homogenize the data, eliminating technical differences such as frame rates and recording durations. Extraneous data embedded in the images (text, scale, etc.) were removed via image masking such that the final input included only the 2D image components. The four masked still frame images were then assembled into a novel input format, a two 2 × 2 image montage. To assess the impact of input data (i.e., frame) selection on model performance, we created two montage formats, Type 1 and Type 2, with frames selected at various points in the cardiac cycle. Given that multiple points in the cardiac cycle are likely more informative than any given still frame, we a priori selected Type 1 to include four frames in a single cardiac cycle approximating the peak R wave, end of T wave, midway through the T-P segment, and beginning of the P wave. Specifically, for each video clip, two consecutive R wave peaks were identified from the ECG tracing. While R waves were easily identifiable in all included images, other waves were less discernible. Thus, to approximate additional points in the cardiac cycle, we divided the R-R interval into halves and thirds and used one-third of the R-R to approximate the end of the T wave, half the R-R to approximate the middle of the T-P segment, and two-thirds of the R-R to approximate the beginning of the P wave. Since it is possible that data from two consecutive cycles may be more informative than data from only one cycle, we created Type 2 montages to include the peak R wave and approximated end of T wave from two consecutive cardiac cycles. Any image coloration was then removed by conversion to grayscale, and the montage was scaled (and zero-padded, as needed) to standardize the dimensions. The final input dimension was 1200 × 900 pixels. Each participant contributed a single Type 1 and Type 2 montage from any given echocardiogram study for model development and testing.

### Data labeling

**Fig. 2:**
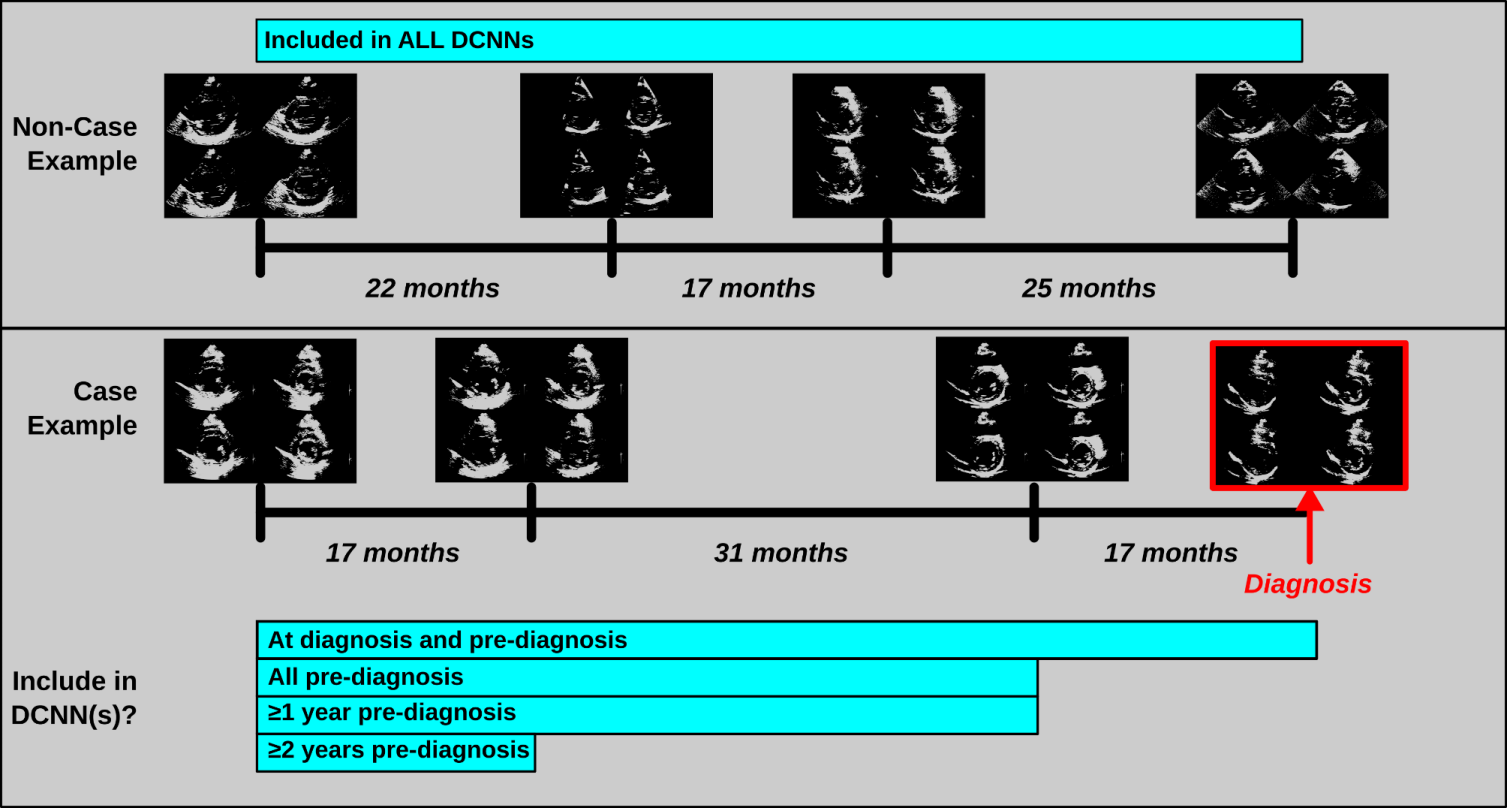
Example of echocardiographic data from non-case and case patients. Interval between echocardiograms for an at-risk non-case and a cardiomyopathy (case) patients displayed in months and index diagnosis shown for the case patient. Four different data groupings for case patient echocardiograms were used to construct the deep convolutional neural networks (DCNNs) based on timing of echocardiogram relative to diagnosis: *at diagnosis and pre-diagnosis* echocardiograms, *pre-diagnosis* echocardiograms, *≥ 1 year pre-diagnosis* echocardiograms, and *≥ 2 years pre-diagnosis* echocardiograms. For this sample case patient, no echocardiograms were performed within 1 year prior to the index diagnosis

To evaluate DCNN quality with varying training dataset sizes, we constructed several sequential models, resampling “case” data by time of echo relative to cardiomyopathy diagnosis, with each sequential model including a smaller pool of echocardiographic data from individuals with present or future cardiomyopathy (Fig. 2) to assess how our model performance deteriorated with increasingly smaller and imbalanced training datasets. We began by building DCNNs with Type 1 and 2 montages from the entire dataset, including all echocardiograms at time of cardiomyopathy diagnosis or pre-diagnosis from individuals with cardiomyopathy and all non-case echocardiograms. For each subsequent DCNN, “case” data were limited to a smaller pool (moving toward a goal of classification at several years prior to cardiomyopathy diagnosis), first removing time of diagnosis data, then limiting the data to at least one- and two-years pre-diagnosis, respectively.

### DCNN architecture and performance

For each of the four model iterations, two separate DCNNs with the same model architecture, one with each montage input type, were run in parallel. The networks were implemented using the Keras (version 2.15) library for Python (version 3.9) with the TensorFlow (version 2.15) backend to construct all DCNNs. The model architecture specifics and hyperparameter details are outlined in Supplementary Table 1 and Supplementary Table 2, respectively. In this case, the sequential network had two principal blocks, each of which contained 2D convolution, batch normalization, and rectified linear unit (ReLU) activation. Batch normalization improves training stability, while ReLU activation introduces non-linearity. The output from the second block was flattened and routed into a fully connected layer, the output of which was passed through ReLU and Softmax layers to produce final estimates of case likelihood for each echocardiogram image montage. The selection of hyperparameters was based on our established DCNN model [14]. A training, validation, and testing split of 70:15:15 was used. Holdout subsets in a 10-fold cross-validation framework were used to test model performance. To prevent data leakage, train-test-validation splits were performed based on patients rather than studies. This ensures that all the data from a given patient is contained within a single split (either train, test, or validation), thereby reducing the risk of information leakage and improving the model’s generalizability. Receiver operating and precision-recall curves were generated for each of the 10 folds, and corresponding area under the curve metrics (area under the receiver operator characteristic curve [AUROC] and area under the precision-recall curve [AUPRC]) were calculated for each fold and averaged to generate mean and standard deviation values. AUPRC is generally favored for assessment of significantly imbalanced datasets [15]. Several additional standard metrics were used to evaluate DCNN performance including F1-score, accuracy, and positive (PPV) and negative (NPV) predictive values, along with ± 1 standard deviation [16]. Type 1 versus Type 2 montage model performance was compared using the combined 5 × 2 fold cross-validation F test [17, 18].

## Results

The analytic dataset included 542 pairs of montages with PSAX echocardiogram frames extracted from 542 unique longitudinal echo studies from 115 at-risk childhood cancer survivors. We were unable to assemble montages due to ECG gating (absent or uninterpretable ECG, single cardiac cycle duration) in 14 studies. Of the 542 montage pairs, 171 were from 45 cases with newly diagnosed or future cardiomyopathy (CM+) and 371 were from 70 non-cases without cardiomyopathy during follow-up (CM–). Median age at cancer diagnosis was 7.0 (Q1-Q3: 3.0–12.0) years with median 9.3 (Q1-Q3: 6.4–12.2) years of follow-up since cancer diagnosis. For CM + survivors, median time from cancer diagnosis to cardiomyopathy diagnosis was 5.9 (Q1-Q3: 0.8–10.3) years. Demographics and treatment characteristics of CM + and CM– survivors are shown in Table 1. The survivors were racially and ethnically diverse with at least 35% hailing from minority populations. They represented a contemporarily treated group with 93% diagnosed with cancer after 1999. All survivors were exposed to at least one anthracycline or anthraquinone treatment, and 42% had received chest radiotherapy. Median doxorubicin-equivalent anthracycline/anthraquinone dose was higher in the CM + than the CM– group (250 [Q1-Q3: 180–375] mg/m^2^ versus 186 [Q1-Q3: 125–300] mg/m^2^, *p* = 0.009). Of the images from CM + survivors, 44 were acquired at the time of diagnosis and 127 were acquired at any point before cardiomyopathy diagnosis with 93 of these acquired ≥ 1 year and 67 acquired ≥ 2 years pre-cardiomyopathy diagnosis, respectively.

**Table 1:** Demographic and clinical characteristics

		Cardiomyopathy cases (CM+)	Non- cases (CM–)	*p*-value
Total		45(100)	70(100)	
Gender	Female	15(33)	34(49)	0.11
	Male	30(67)	36(51)	
Race/ethnicity	White non-Hispanic	23(51)	41(59)	0.20
	Black	7(16)	4(6)	
	Asian	5(11)	4(6)	
	Hispanic	3(7)	13(19)	
	More than one race	2(4)	3(4)	
	Unknown	5(11)	5(7)	
Cancer diagnosis	Leukemia	15(33)	32(46)	0.58
	Lymphoma	11(24)	14(20)	
	Sarcoma	10(22)	11(16)	
	Other solid tumors	9(20)	13(19)	
Age at cancer diagnosis	0–4	16(36)	26(37)	0.86
	5–9	11(24)	21(30)	
	10–14	12(27)	16(23)	
	15–20	6(13)	7(10)	
Year of cancer diagnosis	1991–1999	5(11)	3(4)	0.06
	2000–2005	16(36)	13(19)	
	2006–2009	15(33)	30(43)	
	2010–2015	9(20)	24(34)	
Length of follow-up, years	< 10	23(51)	44(63)	0.21
	10+	22(49)	26(37)	
# anthracycline or anthraquinone	1	34(76)	51(73)	0.51
	2	9(20)	18(26)	
	3	2(4)	1(1)	
Doxorubicin-equivalent anthracycline / anthraquinone dose (mg/m^2^)^1^		250 (180–375)	186 (125–300)	0.009
Heart radiotherapy exposure	Yes	20(44)	28(40)	0.63

Values are n (%) or median (Q1 – Q3).

^1^ Calculated using conversion factors of 0.5 for daunorubicin, 3.0 for idarubicin, 0.67 for epirubicin, and 10.0 for mitoxantrone

**Fig. 3:**
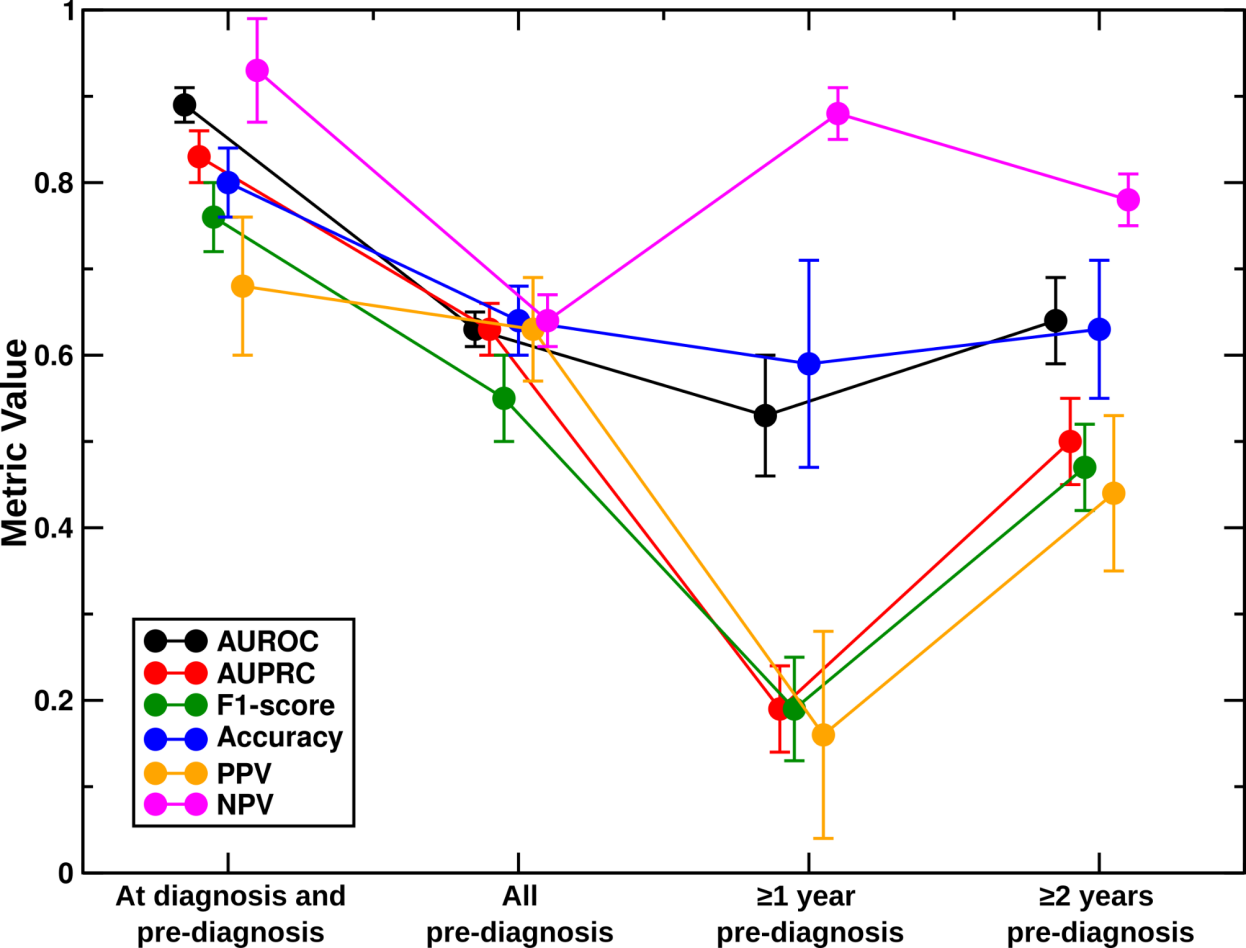
Performance for Type 1 montage convolutional neural networks. As the number and proportion of CM + echocardiograms in the dataset decreased, model performance worsened, with falling area under the precision-recall curve (AUPRC), F1-score, and positive predictive value (PPV). As the dataset imbalance grew, negative predictive value (NPV), area under the receiving operator curve (AUROC), and accuracy, which are less helpful in an imbalanced dataset as they reflect true negatives, changed marginally

Table 2 displays calculated metrics of model performance for each DCNN. Receiver operating and precision-recall curves for all DCNNs are displayed in Supplemental Figs. 1 and 2, respectively. For our complete dataset including all at time of cardiomyopathy diagnosis or pre- diagnosis CM + echocardiograms, the DCNN trained with Type 1 input achieved a mean AUROC = 0.89 ± 0.02, mean AUPRC = 0.83 ± 0.03, F1-score = 0.76 ± 0.04, accuracy = 0.80 ± 0.04, PPV = 0.68 ± 0.08, and NPV = 0.93 ± 0.06. As the dataset grew smaller and more imbalanced, model performance deteriorated with falling AUPRC, F1-score, and PPV (Fig. 3). NPV, AUROC, and accuracy, metrics that reflect true negatives and are thus less helpful in the setting of an imbalanced dataset, changed less as the dataset was limited in size.

**Table 2:** DCNN performance metrics

	CM+ *N*	CM– *N*	AUROC	AUPRC	F1-score	Accuracy	PPV	NPV
*Models including CM + echocardiograms at diagnosis and pre-diagnosis*								
Type 1	171	371	0.89 ± 0.02	0.83 ± 0.03	0.76 ± 0.04	0.80 ± 0.04	0.68 ± 0.08	0.93 ± 0.06
Type 2	171	371	0.88 ± 0.03	0.81 ± 0.05	0.74 ± 0.04	0.79 ± 0.05	0.67 ± 0.07	0.91 ± 0.04
*Models including CM + echocardiograms pre-diagnosis (all)*								
Type 1	127	371	0.63 ± 0.02	0.63 ± 0.03	0.55 ± 0.05	0.64 ± 0.04	0.63 ± 0.06	0.64 ± 0.03
Type 2	127	371	0.64 ± 0.03	0.63 ± 0.04	0.58 ± 0.04	0.63 ± 0.03	0.60 ± 0.04	0.66 ± 0.03
*Models including CM + echocardiograms ≥ 1 year pre-diagnosis*								
Type 1	93	371	0.53 ± 0.07	0.19 ± 0.05	0.19 ± 0.06	0.59 ± 0.12	0.16 ± 0.12	0.88 ± 0.03
Type 2	93	371	0.48 ± 0.09	0.16 ± 0.05	0.16 ± 0.08	0.63 ± 0.08	0.11 ± 0.05	0.88 ± 0.03
*Models including CM + echocardiograms ≥ 2 years pre-diagnosis*								
Type 1	67	371	0.64 ± 0.05	0.50 ± 0.05	0.47 ± 0.05	0.63 ± 0.08	0.44 ± 0.09	0.78 ± 0.03
Type 2	67	371	0.61 ± 0.05	0.48 ± 0.07	0.44 ± 0.10	0.60 ± 0.10	0.41 ± 0.11	0.76 ± 0.05

Performance metrics displayed as average with standard deviations for the 10 folds. AUPRC = area under the precision-recall curve; AUROC = area under the receiver operator characteristic curve; CM + = at-risk survivor who developed cardiomyopathy; CM**–** = at-risk survivor who did not develop cardiomyopathy during follow-up; DCNN = deep convolutional neural network; NPV = negative predictive value; PPV = positive predictive value

Table 3 shows the results of the 5 × 2 fold cross-validation combined F-test, comparing the performance accuracy of the DCNN trained on Type I and Type 2 montages. Performance accuracy was similar between type 1 and Type 2 montage models.

**Table 3:** Performance of type 1 versus 2 montage models

	Type 1 mean accuracy (difference from Type 2 montage model)	F statistic	*p*-value
At diagnosis and pre-diagnosis	0.82(-0.05)	1.78	0.27
Exclusively pre-diagnosis	0.64(-0.03)	1.32	0.40
≥ 1 year pre-diagnosis	0.60(-0.02)	0.66	0.73
≥ 2 years pre-diagnosis	0.60(-0.02)	0.88	0.60

## Discussion

In this feasibility study, we leveraged an image dataset from children and young adults at-risk for cardiomyopathy due to exposure to cardiotoxic cancer therapy to develop a machine learning-driven tool for cardiomyopathy prediction in childhood cancer survivors. Using available data, we built and tested four pairs of DCNNs, sequentially limiting the dataset by timing of echocardiogram from any time point up to and including cardiomyopathy diagnosis to greater than two years before cardiomyopathy diagnosis. Each pair consisted of two DCNNs with differing image data input types (2 × 2 montages of still frames from one versus two cardiac cycles). The performance results with 10-fold cross-validation of our initial model, tasked with differentiating all at time of cardiomyopathy diagnosis or pre-diagnosis case echocardiograms versus non-cases, was reasonable for a feasibility model, with mean AUROC = 0.89 ± 0.02, mean AUPRC = 0.83 ± 0.03, and F1-score = 0.76 ± 0.04 for Type 1 montages. Combined 5 × 2 cross-validation F test found similar performance accuracy with Type 1 versus Type 2 montage input.

Our DCNN-based models’ performances were similar to an existing clinical model developed by Chow et al., which achieved an AUROC of 0.74 using readily available clinical and demographic information (sex, age at cancer diagnosis, and anthracycline and radiotherapy doses) for cumulative heart failure risk through 50 years of age [19, 20]. To our knowledge there are no individual cardiomyopathy risk calculators that incorporate conventional echocardiogram measurements for pediatric cancer patients. Ehrhardt et al. evaluated the utility of adding global longitudinal strain and N-terminal-pro-B-type natriuretic peptide to a standard clinical model in predominantly adult-aged survivors of childhood cancer (median age 37.6 yrs) with normal LV EF for future cardiomyopathy prediction and found that the combined models achieved AUROCs 0.70 to 0.74, not dissimilar to our results [21].

While the number of echocardiograms in this study was limited, to our knowledge, this is the first study using machine learning to predict cardiomyopathy directly from echocardiogram images in pediatric cancer survivors. Machine learning has been applied to other data types for cardiomyopathy prediction in pediatric cancer survivors. Güntürkün et al. applied XGBoost to clinical and ECG data to predict cardiomyopathy in the St. Jude Lifetime Study cohort (*n* > 1000 including 117 cases) with AUROC of 0.89 (95% CI: 0.86 to 0.91) [22]. This was a single institution study with older participants (median age 31.7 years at time of baseline assessment), and results may be less generalizable to younger at-risk survivors of childhood cancer, who typically start cardiomyopathy surveillance within five years of therapy completion [7, 23, 24]. Chaix et al. applied random forest modeling to genetic and clinical data from 289 survivors (183 cases) recruited across six North American centers to develop a prediction model for cardiomyopathy with AUROC 0.72 (95% CI: 0.63 to 0.80) [25], similar discrimination as what we achieved with our DCNN-based example. However, routine genetic sequencing of patients for potential cardiomyopathy–associated variants is not currently standard of care. 2D echocardiography, by contrast, is the recommended screening modality for moderate and high-risk childhood cancer survivors (lifelong echocardiogram screening every two to five years) [7, 23, 24].

Machine learning algorithms have been developed for automated echocardiographic assessment of ventricular function and applied to pediatric echocardiography including EchoNet-Peds and TOMTEC’s AutoLV and AutoSTRAIN [26, 27]. While automated assessment of LV size, geometry, and function may certainly be useful in clinically caring for our patient population, our goal is to predict cardiomyopathy before changes in these parameters of ventricular function are identifiable. To our knowledge, there are no published echocardiogram-based algorithms for pediatric cardiomyopathy disease-state prediction or identification. This is likely due to several challenges: (1) we are unaware of any public use echocardiogram DICOM file datasets specific to pediatric cardiomyopathy patients, (2) echocardiographic data labeling and preprocessing is laborious, and (3) pediatric cardiovascular disease states are rare leading to inherent dataset imbalance. To overcome these obstacles, we plan to update our models with a much larger dataset (> 3,000 echocardiograms and > 100 cardiomyopathy cases) and anticipate that a traditional single-modal machine learning model will be able to ‘see’ myocardial differences on echocardiographic images less obvious to the human eye that may be important predictors of cardiomyopathy risk. We will also explore the use of multiple views (e.g. PSAX and apical four-chamber) and multimodal data fusion techniques to combine echocardiographic data and clinical exposures to see if this enhances predictive capability. Much like clinician learning and decision making, we anticipate that if the DCNN can draw upon both imaging (as in this study) and additionally electronic medical record data, this will enhance performance [21]. Multimodal machine learning is a rapidly evolving field, and the optimal data fusion strategy is unknown [28].

We anticipate that our model could have important clinical implications. Accurate DCNN-based prediction of future cardiomyopathy in *x* years may lead to increased screening frequency and potentially earlier diagnosis, treatment, and improved outcome for high-risk patients. Conversely, it might also prompt consideration of less frequent screening for lower-risk patients, which could be very impactful in the case of families for whom the cost and inconvenience of echocardiography appointments is burdensome, such as those who reside in rural or underprivileged communities. Additionally, patients identified as high-risk may be good candidates for prophylactic therapy such as carvedilol, an investigational medication for prevention of cardiac remodeling in pediatric cancer survivors [29].

## Study limitations

This analysis was intended as a proof of concept and has some limitations. We used an existing dataset where cardiomyopathy was predefined by reduced LV EF or SF cut-offs. Recent guidelines favor EF over SF for cardiomyopathy surveillance [7]. However, in practice, SF is still commonly used to assess LV systolic function in this setting, especially for individuals whose acoustic windows preclude adequate endocardial border delineation for EF assessment. In general, SF remains an accepted quantitative measure of LV systolic function in the pediatric population [30]. SF and EF were measured by the local care teams, and, thus, ground truth for the dataset was subject to inter-rater variability (i.e., some “normal” SF and EF measurements may have been abnormal in the hands of a different cardiologist). Additionally, ‘non-case’ participants were childhood cancer survivors at-risk for cardiomyopathy and may have had subtle cardiac changes not detectable to the human eye, further confounding our ground truth labels. However, we attempted to minimize misclassification by requiring non-cases to have SF ≥ 30% and EF ≥ 55% on all echocardiograms. Non-cases also could not have had any institutionally reported qualitative changes concerning for cardiomyopathy. Any residual misclassification would have introduced conservative bias, reducing the accuracy of our models. If longitudinal echocardiographic data on healthy children were available, discriminatory accuracy may be improved.

Other limitations include that our model input was limited to a single LV view, the PSAX view at the level of the papillary muscles. Given the model was not provided views of all LV wall segments, it may not have been fully exposed to regional wall motion abnormalities. We plan to address this in future model iterations by adding the apical four-chamber view. The amount of echocardiographic data available in this initial study also was small, and there was an imbalance of cases and non-cases, which limits DCNN performance. As anticipated, with increasingly limited dataset size, DCNN performance deteriorated with worsening AUPRC, F1-score, and PPV. Limited dataset size can be a challenge in pediatric studies of rare but serious outcomes, and the feasibility of machine learning for disease classification tasks in pediatric cardiology is largely unknown. Encouraged by the modest success of our feasibility model, we are now working with sites across COG to gather additional cardiomyopathy cases, which should allow us to bolster model performance and improve generalizability. Another limitation of this study is the empirical choice of creating montages using specific frames from the cardiac cycle rather than using multiple frames as input to the DCNN. This decision was intended to reduce computational complexity. In future work, we plan to implement down-sampling strategies to reduce frame dimensions, enabling the use of multiple frames to capture temporal features from echocardiogram clips through the application of 3D DCNNs.

## Conclusions

Our results suggest that a DCNN tasked with classifying cardiomyopathy status with real world pediatric echocardiographic data is feasible. A model capable of predicting which at-risk childhood cancer survivors will develop cardiomyopathy in the future will be a clinically useful tool for routine echocardiography surveillance, helping physicians to identify and potentially modify risk for survivors at the highest risk for cardiomyopathy.

## Electronic supplementary material

Below is the link to the electronic supplementary material.

Supplementary Material 1

Supplementary Material 2

Supplementary Material 3

Supplementary Material 4

Supplementary Material 5

## Data Availability

The datasets generated and/or analyzed during the current study are not publicly available due to their sensitive nature. Source code will be made publicly available upon project completion.

## Declarations

### Authors’ information

The preliminary results were presented in part by CY at the 1st International Pediatric Cardio-Oncology meeting in October 2022 in Cincinnati, Ohio.

## Abbreviations

2D: 2-dimensional
AUPRC: Area under the precision-recall curve
AUROC: Area under the receiver operator characteristic curve
COG: Children’s Oncology Group
DCNN: Deep convolutional neural network
ECG: Electrocardiogram
EF: Ejection fraction
LV: Left ventricular
NPV: Negative predictive value
PPV: Positive predictive value
PSAX: Parasternal short axis
SF: Shortening fraction

## References

[1] Lipshultz SE, Adams MJ, Colan SD, Constine LS, Herman EH, Hsu DT, et al. Long-term cardiovascular toxicity in children, adolescents, and young adults who receive cancer therapy: pathophysiology, course, monitoring, management, prevention, and research directions: a scientific statement from the American Heart Association. Circulation. 2013;128:1927–95. 24081971 10.1161/CIR.0b013e3182a88099

[2] Bottinor W, Im C, Doody DR, Armenian SH, Arynchyn A, Hong B, et al. Mortality after major cardiovascular events in survivors of childhood cancer. J Am Coll Cardiol. 2024;83:827–38. 38383098 10.1016/j.jacc.2023.12.022 PMC11144450

[3] Cardinale D, Colombo A, Lamantia G, Colombo N, Civelli M, De Giacomi G, et al. Anthracycline-induced cardiomyopathy: clinical relevance and response to pharmacologic therapy. J Am Coll Cardiol. 2010;55:213–20. 20117401 10.1016/j.jacc.2009.03.095

[4] Investigators SOLVD, Yusuf S, Pitt B, Davis CE, Hood WB, Cohn JN. Effect of enalapril on mortality and the development of heart failure in asymptomatic patients with reduced left ventricular ejection fractions. N Engl J Med. 1992;327:685–91. 1463530 10.1056/NEJM199209033271003

[5] Pfeffer MA, Braunwald E, Moyé LA, Basta L, Brown EJ, Cuddy TE, et al. Effect of captopril on mortality and morbidity in patients with left ventricular dysfunction after myocardial infarction. Results of the survival and ventricular enlargement trial. The SAVE investigators. N Engl J Med. 1992;327:669–77. 1386652 10.1056/NEJM199209033271001

[6] Mertens L, Singh G, Armenian S, Chen M-H, Dorfman AL, Garg R, et al. Multimodality imaging for cardiac surveillance of cancer treatment in children: recommendations from the American Society of Echocardiography. J Am Soc Echocardiogr. 2023;36:1227–53. 38043984 10.1016/j.echo.2023.09.009

[7] Ehrhardt MJ, Leerink JM, Mulder RL, Mavinkurve-Groothuis A, Kok W, Nohria A, et al. Systematic review and updated recommendations for cardiomyopathy surveillance for survivors of childhood, adolescent, and young adult cancer from the International Late effects of Childhood Cancer Guideline Harmonization Group. Lancet Oncol. 2023;24:e108–20. 37052966 10.1016/S1470-2045(23)00012-8

[8] Ramjaun A, AlDuhaiby E, Ahmed S, Wang L, Yu E, Nathan PC, et al. Echocardiographic detection of cardiac dysfunction in childhood cancer survivors: how long is screening required? Pediatr Blood Cancer. 2015;62:2197–203. 26146944 10.1002/pbc.25651 PMC4670474

[9] Lipshultz SE, Lipsitz SR, Sallan SE, Simbre VC, Shaikh SL, Mone SM, et al. Long-term enalapril therapy for left ventricular dysfunction in doxorubicin-treated survivors of childhood cancer. J Clin Oncol. 2002;20:4517–22. 12454107 10.1200/JCO.2002.12.102

[10] Border WL, Sachdeva R, Stratton KL, Armenian SH, Bhat A, Cox DE, et al. Longitudinal changes in echocardiographic parameters of cardiac function in pediatric cancer survivors. JACC CardioOncol. 2020;2:26–37. 32719829 10.1016/j.jaccao.2020.02.016 PMC7384713

[11] Harris PA, Taylor R, Thielke R, Payne J, Gonzalez N, Conde JG. Research electronic data capture (REDCap)—A metadata-driven methodology and workflow process for providing translational research informatics support. J Biomed Inform. 2009;42:377–81. 18929686 10.1016/j.jbi.2008.08.010 PMC2700030

[12] Harris PA, Taylor R, Minor BL, Elliott V, Fernandez M, O’Neal L, et al. The REDCap consortium: building an international community of software platform partners. J Biomed Inf. 2019;95:103208. 10.1016/j.jbi.2019.103208 PMC7254481 31078660

[13] Lopez L, Saurers DL, Barker PCA, Cohen MS, Colan SD, Dwyer J, et al. Guidelines for performing a Comprehensive Pediatric Transthoracic Echocardiogram: recommendations from the American Society of Echocardiography. J Am Soc Echocardiogr. 2024;37:119–70. 38309834 10.1016/j.echo.2023.11.015

[14] Sridhar AR, Chen Amber Z-H, Mayfield JJ, Fohner AE, Arvanitis P, Atkinson S, et al. Identifying risk of adverse outcomes in COVID-19 patients via artificial intelligence-powered analysis of 12-lead intake electrocardiogram. Cardiovasc Digit Health J. 2022;3:62–74. 35005676 10.1016/j.cvdhj.2021.12.003 PMC8719367

[15] Davis J, Goadrich M. The relationship between Precision-Recall and ROC curves. Proceedings of the 23rd international conference on Machine Learning - ICML ’06. Pittsburgh, Pennsylvania: ACM Press; 2006 [cited 2024 Jan 23]. pp. 233–40. http://portal.acm.org/citation.cfm?doid=1143844.1143874

[16] Powers DMW. Evaluation: from precision, recall and F-measure to ROC, informedness, markedness and correlation. arXiv; 2020 [cited 2024 Jan 24]. http://arxiv.org/abs/2010.16061

[17] Alpaydin E. Combined 5 x 2 cv F test for comparing supervised classification learning algorithms. Neural Comput. 1999;11:1885–92. 10578036 10.1162/089976699300016007

[18] Basu S, Hall LO, Goldgof DB, Gu Y, Kumar V, Choi J et al. Developing a classifier model for lung tumors in CT-scan images. 2011 IEEE International Conference on Systems, Man, and Cybernetics. 2011 [cited 2024 Mar 19]. pp. 1306–12. https://ieeexplore.ieee.org/document/6083840

[19] Chow EJ, Chen Y, Kremer LC, Breslow NE, Hudson MM, Armstrong GT, et al. Individual prediction of heart failure among childhood cancer survivors. J Clin Oncol. 2015;33:394–402. 25287823 10.1200/JCO.2014.56.1373 PMC4314592

[20] Chow EJ, Chen Y, Hudson MM, Feijen EAM, Kremer LC, Border WL, et al. Prediction of Ischemic Heart Disease and Stroke in survivors of Childhood Cancer. J Clin Oncol. 2018;36:44–52. 29095680 10.1200/JCO.2017.74.8673 PMC5756324

[21] Ehrhardt MJ, Liu Q, Mulrooney DA, Rhea IB, Dixon SB, Lucas JT et al. Improved cardiomyopathy risk prediction using global longitudinal strain and N-terminal-pro-B-type natriuretic peptide in survivors of childhood cancer exposed to cardiotoxic therapy. J Clin Oncol. 2024;JCO2301796. 10.1200/JCO.23.01796 PMC11095874 38207238

[22] Güntürkün F, Akbilgic O, Davis RL, Armstrong GT, Howell RM, Jefferies JL et al. Artificial intelligence–assisted prediction of late-onset cardiomyopathy among childhood cancer survivors. JCO Clin Cancer Inf. 2021;5:CCI.20.00176. 10.1200/CCI.20.00176 PMC8462657 33909450

[23] Armenian SH, Hudson MM, Mulder RL, Chen MH, Constine LS, Dwyer M, et al. Recommendations for cardiomyopathy surveillance for survivors of childhood cancer: a report from the International Late effects of Childhood Cancer Guideline Harmonization Group. Lancet Oncol. 2015;16:e123–136. 25752563 10.1016/S1470-2045(14)70409-7 PMC4485458

[24] Children’s Oncology Group. Long-term follow-up guidelines for survivors of childhood, adolescent, and young adult cancers, Version 6. www.survivorshipguidelines.org. 2023 [cited 2024 Jan 16]. http://survivorshipguidelines.org/pdf/2023/COG_LTFU_Guidelines_Comprehensive_v6.pdf

[25] Chaix M-A, Parmar N, Kinnear C, Lafreniere-Roula M, Akinrinade O, Yao R, et al. Machine learning identifies clinical and genetic factors associated with anthracycline cardiotoxicity in pediatric cancer survivors. JACC CardioOncol. 2020;2:690–706. 34396283 10.1016/j.jaccao.2020.11.004 PMC8352204

[26] Reddy CD, Lopez L, Ouyang D, Zou JY, He B. Video-based deep learning for automated assessment of left ventricular ejection fraction in pediatric patients. J Am Soc Echocardiogr. 2023;36:482–9. 36754100 10.1016/j.echo.2023.01.015

[27] Li L, Homer P, Craft M, Kutty S, Putschoegl A, Marshall A et al. Machine learning-enabled fully automated assessment of left ventricular volume, ejection fraction and strain: Experience in pediatric and young adult echocardiography. Pediatr Cardiol. 2022 [cited 2024 Jan 16]; 10.1007/s00246-022-03015-7 10.1007/s00246-022-03015-7 36208311

[28] Mohsen F, Ali H, El Hajj N, Shah Z. Artificial intelligence-based methods for fusion of electronic health records and imaging data. Sci Rep. 2022;12:17981. 36289266 10.1038/s41598-022-22514-4 PMC9605975

[29] Armenian SH, Hudson MM, Lindenfeld L, Chen S, Chow EJ, Colan S et al. Effect of carvedilol versus placebo on cardiac function in anthracycline-exposed survivors of childhood cancer (PREVENT-HF): a randomised, controlled, phase 2b trial. The Lancet Oncology. 2024 [cited 2024 Jan 15];0. https://www.thelancet.com/journals/lanonc/article/PIIS1470-2045(23)00637-X/fulltext 10.1016/S1470-2045(23)00637-X PMC10872217 38215764

[30] Lopez L, Colan SD, Frommelt PC, Ensing GJ, Kendall K, Younoszai AK, et al. Recommendations for quantification methods during the performance of a pediatric echocardiogram: a report from the Pediatric Measurements Writing Group of the American Society of Echocardiography Pediatric and Congenital Heart Disease Council. J Am Soc Echocardiogr. 2010;23:465–95. 20451803 10.1016/j.echo.2010.03.019

