# Supplementary figures and images for: Building a machine learning-assisted echocardiography prediction tool for children at risk for cancer therapy-related cardiomyopathy
Source: Cardiooncology. 2024 Oct 9;10:66. doi: 10.1186/s40959-024-00268-4 (PMC11462765; doi:10.1186/s40959-024-00268-4)

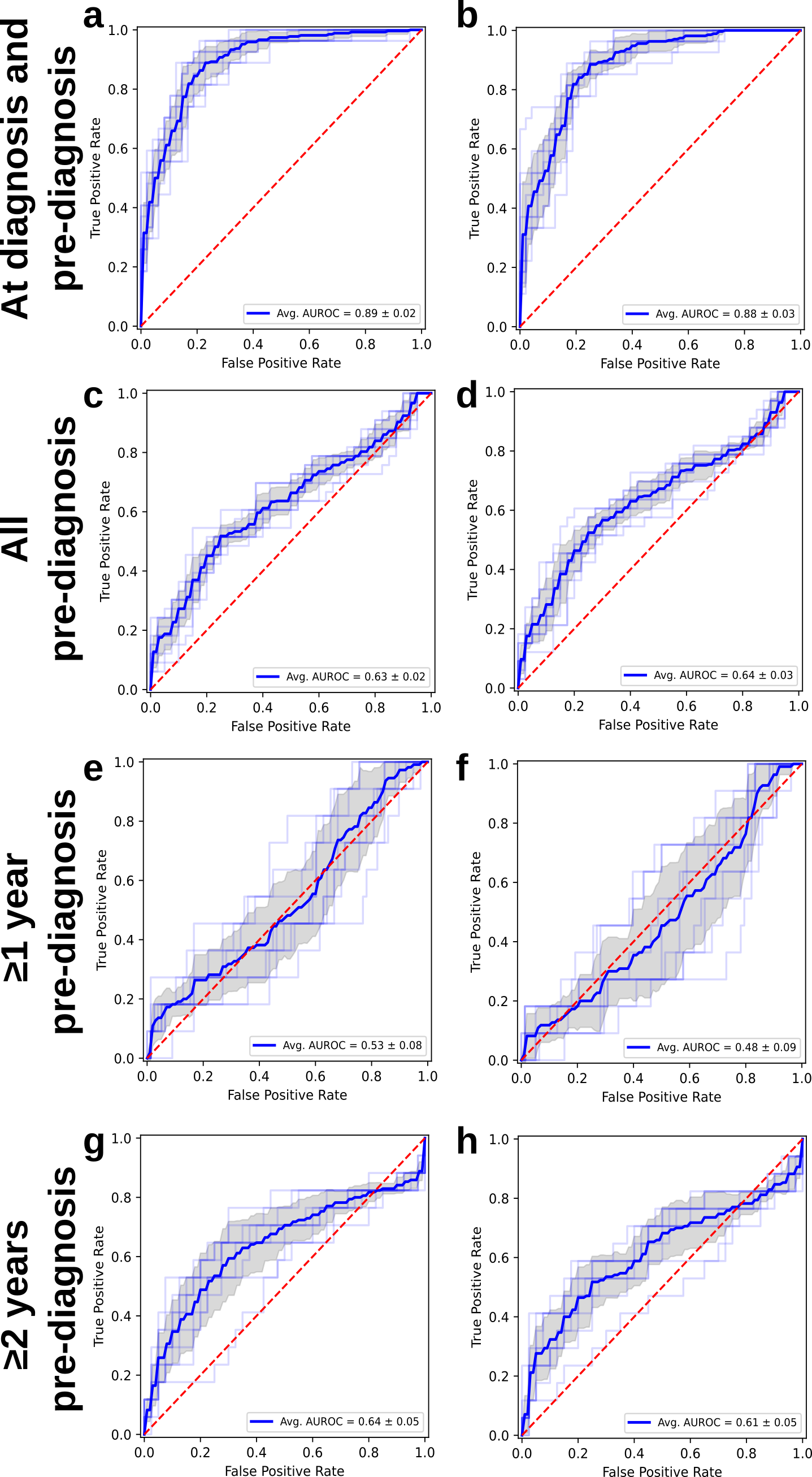

Supplement: Supplementary file 1 — Supplementary Material 1 [file 40959_2024_268_MOESM1_ESM.jpeg]

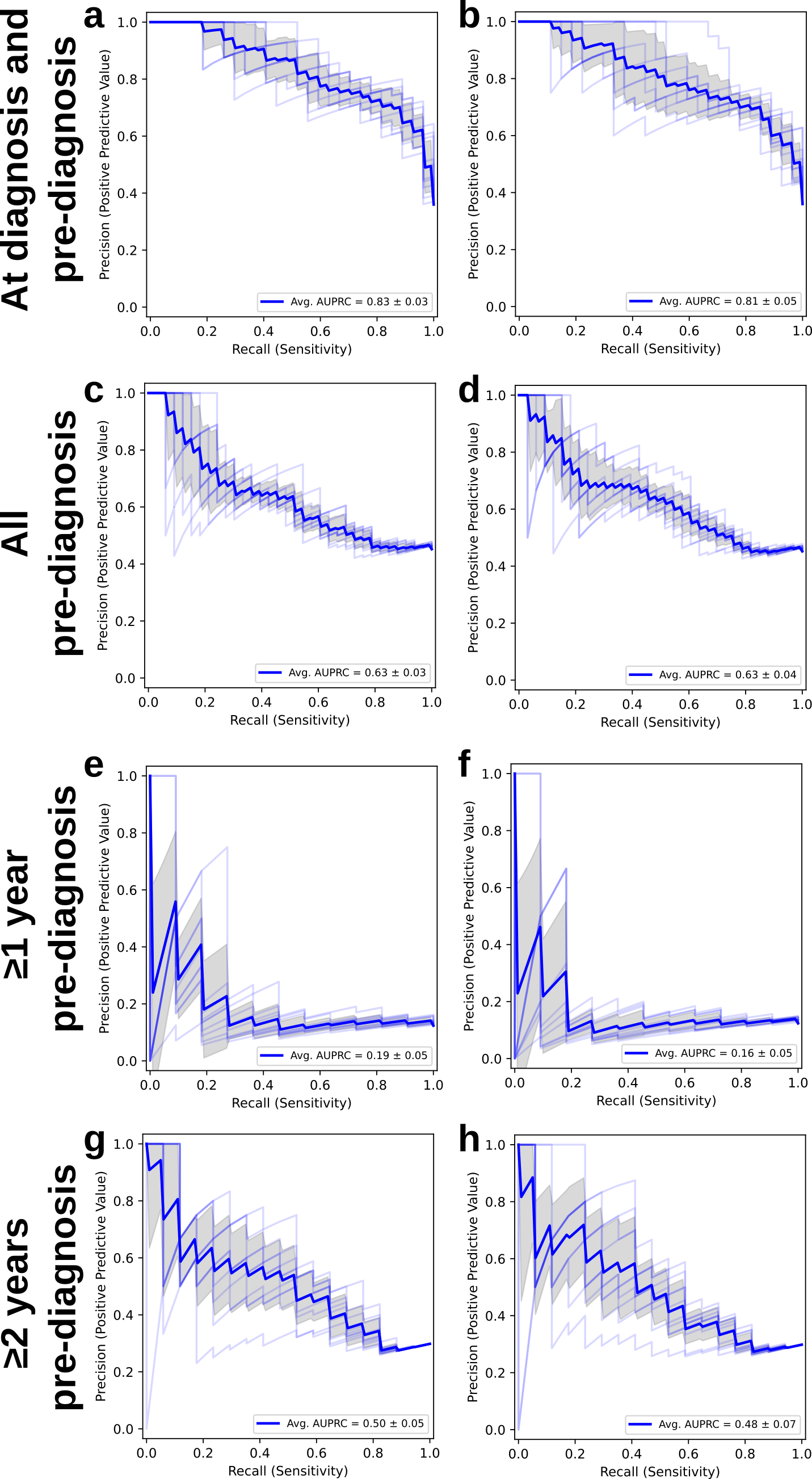

Supplement: Supplementary file 2 — Supplementary Material 2 [file 40959_2024_268_MOESM2_ESM.jpeg]
