# Supplementary material for: Building a machine learning-assisted echocardiography prediction tool for children at risk for cancer therapy-related cardiomyopathy
Source: Cardiooncology. 2024 Oct 9;10:66. doi: 10.1186/s40959-024-00268-4 (PMC11462765; doi:10.1186/s40959-024-00268-4)
Supplement: Supplementary file 3 — Supplementary Material 3 [file 40959_2024_268_MOESM3_ESM.docx]

| **Supplementary Table 1. DCNN model architecture for input shape = [800,800]** | | |
| --- | --- | --- |
| **Layer** | **Output Shape** | **Description** |
| **Convolution Layer 1** | | |
| Conv2D | [399,399,16] | 16 2D feature maps |
| Batch Normalization | [399,399,16] | Normalize feature maps |
| ReLU | [399,399,16] | Introduce non-linearity |
|  | **Convolution Layer 2** |  |
| Conv2D | [199,199,16] | 16 2D feature maps |
| Batch Normalization | [199,199,16] | Normalize feature maps |
| ReLU | [199,199,16] | Introduce non-linearity |
|  | **Output Layer** |  |
| Flatten | [633616] | Reshape to vector |
| Dense | [64] | Feature aggregation |
| Dense + Sigmoid | [2] | Logit to output |
