# Supplementary material for: Building a machine learning-assisted echocardiography prediction tool for children at risk for cancer therapy-related cardiomyopathy
Source: Cardiooncology. 2024 Oct 9;10:66. doi: 10.1186/s40959-024-00268-4 (PMC11462765; doi:10.1186/s40959-024-00268-4)
Supplement: Supplementary file 4 — Supplementary Material 4 [file 40959_2024_268_MOESM4_ESM.docx]

| **Supplementary Table 2. Hyperparameters for DCNN model** | |
| --- | --- |
| **Number of epochs** | 100 |
| **Batch size** | 32 |
| **Learning rate** | 0.00001 |
| **Optimizer** | Adam’s optimizer |
