# Supplementary material for: Building a machine learning-assisted echocardiography prediction tool for children at risk for cancer therapy-related cardiomyopathy
Source: Cardiooncology. 2024 Oct 9;10:66. doi: 10.1186/s40959-024-00268-4 (PMC11462765; doi:10.1186/s40959-024-00268-4)
Supplement: Supplementary file 5 — Supplementary Material 5 [file 40959_2024_268_MOESM5_ESM.docx]

**Supplementary figure legends**

**Supplementary Figure 1. AUROC for cardiomyopathy classification with Type 1 input montages (a,c,e,g) and Type 2 input montages (b,d,f,h).**

**Supplementary Figure 2. AUPRC for cardiomyopathy classification with Type 1 input montages (a,c,e,g) and Type 2 input montages (b,d,f,h).**
